# Nano‐selenium relieved hepatic and renal oxidative damage in hypothyroid rats

**DOI:** 10.14814/phy2.15682

**Published:** 2023-05-05

**Authors:** Mahmoud Hosseini, Farimah Behehsti, Narges Marefati, Akbar Anaeigoudari

**Affiliations:** ^1^ Psychiatry and Behavioral Sciences Research Center Mashhad University of Medical Sciences Mashhad Iran; ^2^ Neuroscience Research Center Torbat Heydariyeh University of Medical Sciences Torbat Heydariyeh Iran; ^3^ Department of Physiology, School of Paramedical Sciences Torbat Heydariyeh University of Medical Sciences Iran; ^4^ Applied Biomedical Research Center Mashhad University of Medical Sciences Mashhad Iran; ^5^ Department of Physiology, School of Medicine Jiroft University of Medical Sciences Jiroft Iran

**Keywords:** hepatic, hypothyroidism, nano‐selenium, oxidative stress, renal

## Abstract

Hypothyroidism can induce oxidative stress. Nano‐selenium (Nano Sel) has antioxidant effects. The current research explored Nano Sel effects on hepatic and renal oxidative damage induced by hypothyroidism in rats. Animals were grouped into (1) Control; (2) Propylthiouracil (PTU) group which received water mixed with 0.05% of PTU; (3) PTU‐Nano Sel 50; (4) PTU‐Nano Sel 100; and (5) PTU‐Nano Sel 150. Besides PTU, the PTU‐Nano Sel groups were treated with 50, 100, or 150 μg/kg of Nano Sel intraperitoneally. Treatments were done for 6 weeks. The serum level of T4, aspartate transaminase (AST), alanine transaminase (ALT), alkaline phosphatase (ALP), albumin, total protein, creatinine, and blood urea nitrogen (BUN) was evaluated. Malondialdehyde (MDA) and total thiol concentration and the activity of catalase (CAT) and superoxide dismutase (SOD) in hepatic and renal tissues also were checked. Hypothyroidism induced by PTU significantly increased AST, ALT, ALP, creatinine, BUN, and MDA concentration and noticeably reduced albumin, total protein, total thiol level, and SOD and CAT activity. Administration of Nano Sel ameliorated the adverse effects of hypothyroidism on liver and kidney function. Nano Sel applied protective effects against hepatic and renal damage resulting from hypothyroidism via ameliorating the oxidative stress status. More cellular and molecular experiments need to be done to understand the exact mechanisms.

## Graphical abstract

Hypothyroidism caused by propylthiouracil (PTU) resulted in liver and renal dysfunction through inducing oxidative stress. Treatment by Nano‐selenium improved the harmful impacts of hypothyroidism on liver and renal function by modulation of oxidative stress.

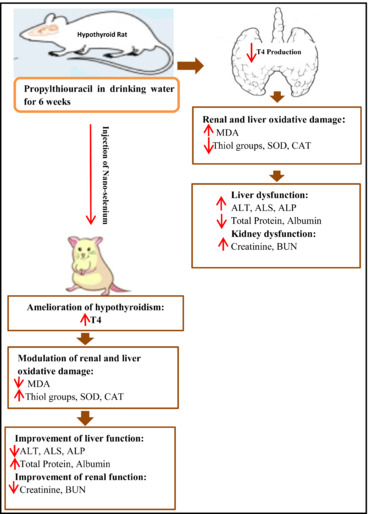

## 1 INTRODUCTION

Thyroid hormones regulate the rate of basal metabolism by adjusting the oxygen utilization of tissues (Khakisahneh et al., 2019). A large number of people in the world suffer from thyroid gland disturbances (Das et al., 2022). Among the different types of thyroid disturbances, hypothyroidism is more common than others. Hypothyroidism is defined as a condition in which thyroid hormones are synthesized in a lesser amount than the normal state (Larsen, 1982). Hypothyroidism can be associated with the uncontrolled release of inflammatory cytokines and reactive species oxygen (ROS) and consequently causes oxidative damage to body organs (Nanda et al., 2011). It has been suggested that hypothyroidism‐linked oxidative damage is a consequence of both the overproduction of free radicals and a reduced level of antioxidant defense (Nanda et al., 2008). In addition, mitochondrial respiratory chain dysfunction resulting from a decreased level of thyroid hormones increases ROS generation and induces oxidative stress (Resch et al., 2002).

Selenium (Se) is an essential trace mineral that has both organic and inorganic forms (Kieliszek et al., 2021). Selenocysteine (SeCys) and selenomethionine (SeMet) are considered organic forms of Se whereas elemental Se, selenite, selenate, and selenide are its inorganic forms (Kieliszek, 2019). The main form of dietary Se for humans is inorganic form. Selenoproteins (SePs) are a large group of Se‐containing proteins that play important biological roles (Avery & Hoffmann, 2018). These proteins protect the cells against the harmful effects of ROS (Zhang et al., 2020). Glutathione peroxidase (GPx) also is the first SeP discovered that acts as a potent antioxidant enzyme in the cells (Ighodaro & Akinloye, 2018). In addition, Se performs a basic role in lipid metabolism, and Se defect can disturb the plasma lipid profile (Xu et al., 2017). Along with this evidence, the results of some studies show that Se supplementations improved the detrimental effects of toxins on the immune system and antioxidant defense (Khazraei et al., 2021). Therefore, the use of selenium nanoparticles (SeNPs) is preferred to Se (Talebi et al., 2017). Considering these backgrounds, the current study aimed to evaluate nano‐selenium (Nano Sel) effects on hepatic and renal oxidative damage induced by hypothyroidism in rats.

## 2 MATERIALS AND METHODS

### 2.1 Animals and groups

In this research, 40 male Wistar rats prepared from the animal nest of Mashhad University of Medical Sciences, Mashhad, Iran, were distributed into five groups: Group 1 (Control) received water. Group 2 (PTU) was treated with 0.05% propylthiouracil (PTU)‐containing water (Subudhi & Chainy, 2012). These two groups also received saline intraperitoneally. Group 3 (PTU‐Nano Sel50), Group 4 (PTU‐Nano Se100), and Group 5 (PTU‐Nano Sel150) received 0.05% PTU‐containing water and were treated, respectively, by 50, 100, and 150 μg/kg/day of Nano Sel intraperitoneally. The rats were kept under a constant temperature (22 ± 2°C) and a 12‐h light/dark cycle along with sufficient water and food. All experimental procedures were carried out based on the guidelines and instructions described by the Ethical Committee of Jiroft University of Medical Sciences, Jiroft, Iran (IR.JMU.REC.1400.068). Treatments were done for 6 weeks. Hypothyroidism induced by PTU was checked by collecting the blood samples and measuring the level of free thyroxin (fT4) using the radioimmunoassay method (Diasource, T4‐RIA‐CT).

PTU used in the present study was provided by Sigma (Sigma Aldrich Chemical Co.). Considering the previous studies (Bai et al., 2017; Rastegar moghaddam et al., 2022), Nano Sel was prepared by the Department of Biotechnology of Mashhad University of Medical Sciences and was confirmed by Biotechnology Department.

### 2.2 Measurement of biochemical parameters

#### 2.2.1 Evaluation of liver and kidney function indicators

At the end of 6 weeks, the blood specimens were extracted from the heart of the rats. After centrifuging at 3500 *g* for 15 min, they were employed for assessment of aspartate transaminase (AST), alanine transaminase (ALT), alkaline phosphatase (ALP), albumin, total protein, creatinine, and blood urea nitrogen (BUN).

#### 2.2.2 Measurement of oxidative stress parameters

For assessment of oxidative stress parameters, the liver and kidney tissues of rats were homogenized using phosphate buffer. Then, homogenized tissues were centrifuged (10,000 *g*) for 20 min, and eventually, supernatant was gathered and used.

#### 2.2.3 Estimation of malondialdehyde (MDA)

The level of MDA in liver and kidney tissue homogenates was estimated as described in previous studies (Beheshti et al., 2020). 250 μL of specimens was mixed with an adequate amount of trichloroacetic acid (TCA) and thiobarbituric acid (TBA). Mixtures were bubbled in a water bath. The solutions were cooled, and the absorbance was defined at 532 nm.

#### 2.2.4 Evaluation of total thiol groups

For the determination of total thiol groups, Tris buffer (2 mL, 0.1 M) and 5, 50‐Dithiobis (2‐nitrobenzoic acid) (DTNB) (5 mL) were added to 0.5 mL of samples. After incubating in the dark environment for 30 min, the absorbance of samples was determined at 412 nm (Beheshti et al., 2020).

#### 2.2.5 Assessment of superoxide dismutase (SOD) activity

Assessment of SOD activity in hepatic and renal tissues was done using a colorimetric procedure. In this procedure, superoxide resulting from the auto‐oxidation of pyrogallol converts tetrazolium into a colored compound named formazan. Homogenated samples (0.1 mL), Tris–HCl buffer (2.5 mL), and pyrogallol (0.1 mL) were mixed. The absorbance was monitored at 420 nm (Baghcheghi et al., 2018).

#### 2.2.6 Determination of catalase (CAT) activity

The CAT activity was determined by its ability to break hydrogen peroxide. 0.1 mL of supernatant and 0.1 mL of H_2_O_2_ with a sufficient amount of phosphate buffer were blended. Absorbance was read at 240 nm (Mansouri et al., 2021).

### 2.3 Statistical analysis

In the present study, data were exhibited as mean ± SEM. One‐way ANOVA followed by Tukey's test was performed for data analysis and comparison of groups. *p* < 0.05 is used for a significant level.

## 3 RESULTS

### 3.1 Effect of Nano Sel on the blood level of fT4

**FIGURE 1:**
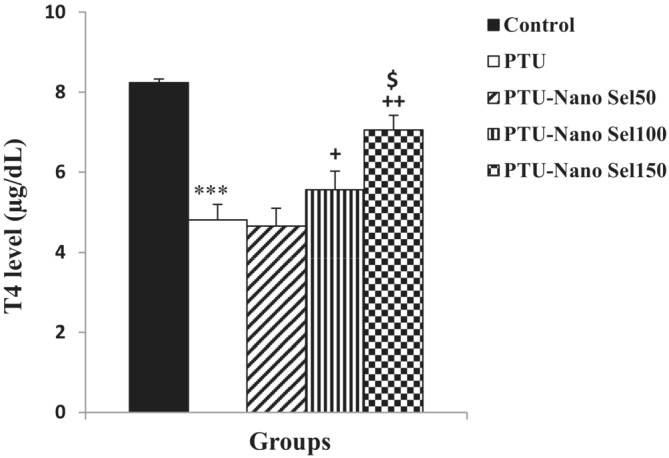
Comparison of the serum level of T4 between experimental groups. Data were displayed as mean ± SEM. ***p < 0.001 versus control group; ^+^ *p* < 0.05 and ^++^ *p* < 0.01 versus PTU group; ^$^ *p* < 0.05 versus Nano Sel50 group.

The effect of Nano Sel on the serum level of fT4 has been presented in Figure 1. As shown, PTU decreased the serum level of fT4 in the PTU group with respect to the control group (*p* < 0.001). Based on the results, the fT4 concentration in PTU‐Nano Sel100 (*p* < 0.05) and PTU‐Nano Sel 150 (*p* < 0.01) groups was higher than the PTU group. The level of fT4 in the Nano Sel150 group also was higher than in the PTU‐Nano Sel50 group (*p* < 0.05).

### 3.2 Effect of Nano Sel on liver function indicators

**FIGURE 2:**
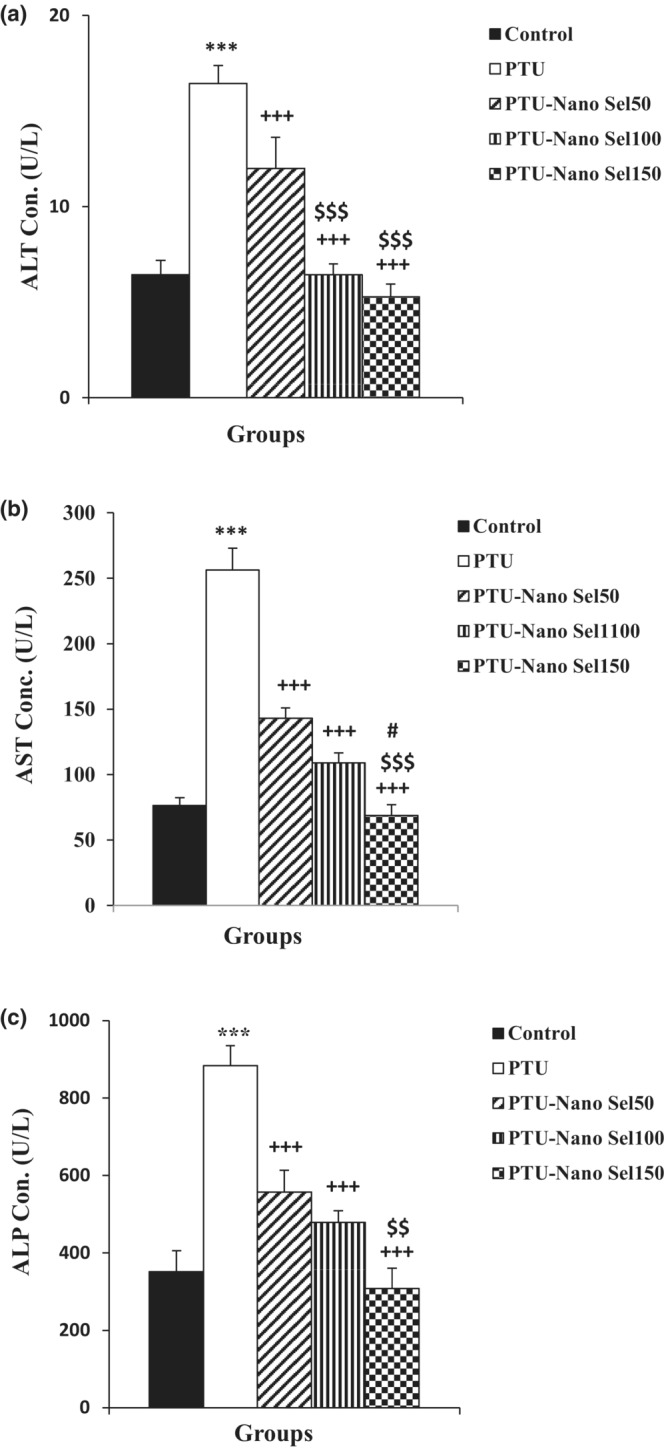
Comparison of the serum level of ALT (a), AST (b) and ALP (c) between experimental groups. Findings were presented as mean ± SEM. ***p < 0.001 versus control group; ^+++^ *p* < 0.001 versus PTU group; ^$$^ *p* < 0.01 and ^$$$^ *p* < 0.001 versus Nano Sel50 group; ^#^ *p* < 0.05 versus Nano Sel100 group.

**FIGURE 3:**
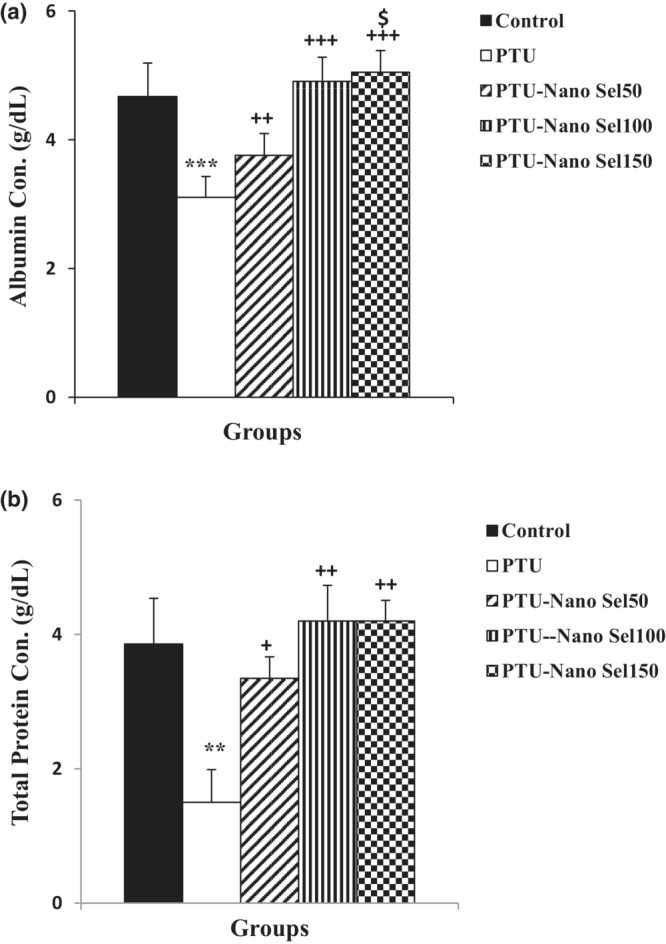
Comparison of the serum level of albumin (a) and total protein (b) between experimental groups. Results were shown as mean ± SEM. **p < 0.01 and ***p < 0.001 versus control group; ^+^ *p* < 0.05, ^++^ *p* < 0.01 and ^+++^ *p* < 0.001 versus PTU group; ^$^ *p* < 0.05 versus Nano Sel50 group; ^#^ *p* < 0.05 versus Nano Sel100 group.

The effect of Nano Sel on liver function indicators has been exhibited in Figures 2 and 3. As specified in Figure 2, the blood concentration of ALT, AST, and ALP is higher in the PTU group versus the control group (*p* < 0.001 for all). Administration of all three doses of Nano Sel decreased the level of ALT, AST, and ALP in PTU‐Nano Sel groups versus the PTU group (*p* < 0.001 for all). The level of ALT, AST, and ALP in the Nano Sel 150 group was lower than in the Nano Sel50 group (*p* < 0.01 and *p* < 0.001). According to Figure 3, the serum level of albumin (*p* < 0.001) and total protein (*p* < 0.01) is lower in the PTU group compared with the control group. The blood concentration of albumin (*p* < 0.01 and *p* < 0.001) and total protein (*p* < 0.05 and *p* < 0.01) increased in PTU‐Nano Sel groups in comparison with the PTU group. In addition, the blood concentration of albumin in the Nano Sel150 group was higher than in the Nano Sel50 group (*p* < 0.05).

### 3.3 Effect of Nano Sel on renal function indicators

**FIGURE 4:**
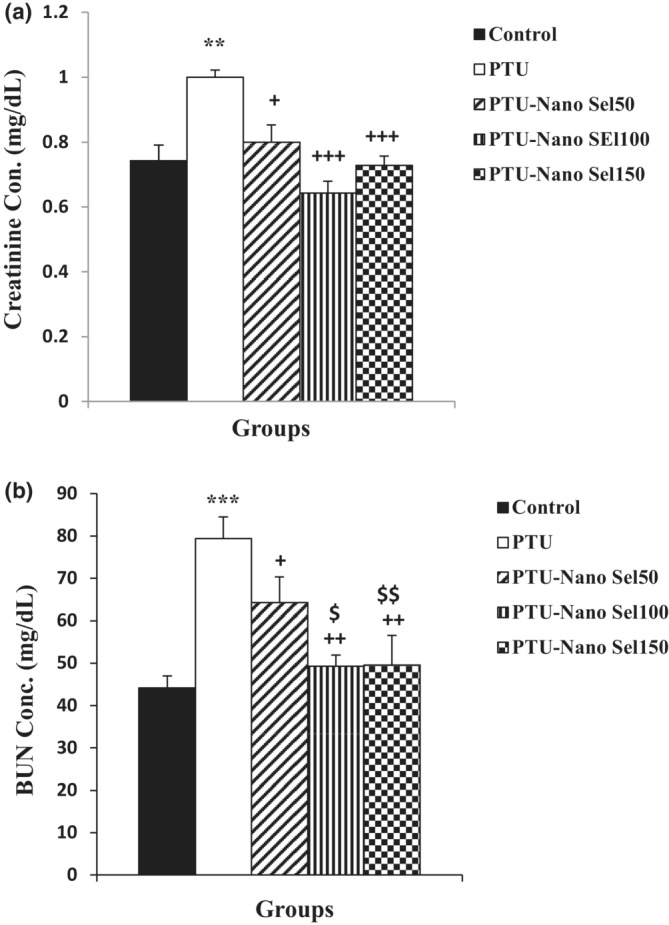
Comparison of the serum level of creatinine (a) and BUN (b) between experimental groups. Results were demonstrated as mean ± SEM. **p < 0.01 and ***p < 0.001 versus control group; ^+^ *p* < 0.05, ^++^ *p* < 0.01 and ^+++^ *p* < 0.001 versus PTU group; ^$^ *p* < 0.05 and ^$$^ *p* < 0.01 versus Nano Sel50 group.

The results of the Nano Sel effect on renal function indexes have been illustrated in Figure 4. As indicated, the serum content of creatinine (*p* < 0.01) and BUN (*p* < 0.001) in rats treated by PTU is higher than those of the control group. Findings revealed that the creatinine concentration reduced in PTU‐Nano Sel50 (*p* < 0.05), PTU‐Nano Sel100 (*p* < 0.001), and PTU‐Nano Sel150 (*p* < 0.001) groups compared with the PTU group. The blood level of BUN also significantly diminished in PTU‐Nano Sel50 (*p* < 0.05), PTU‐Nano Sel100 (*p* < 0.01), and PTU‐Nano Sel150 (*p* < 0.001) groups versus the PTU group. Furthermore, the BUN level in PTU‐Nano Sel100 (*p* < 0.05) and PTU‐Nano Sel150 (*p* < 0.01) groups was higher than in the PTU‐Nano Sel50 group.

### 3.4 Effect of Nano Sel on oxidative stress parameters in liver and renal tissues

**FIGURE 5:**
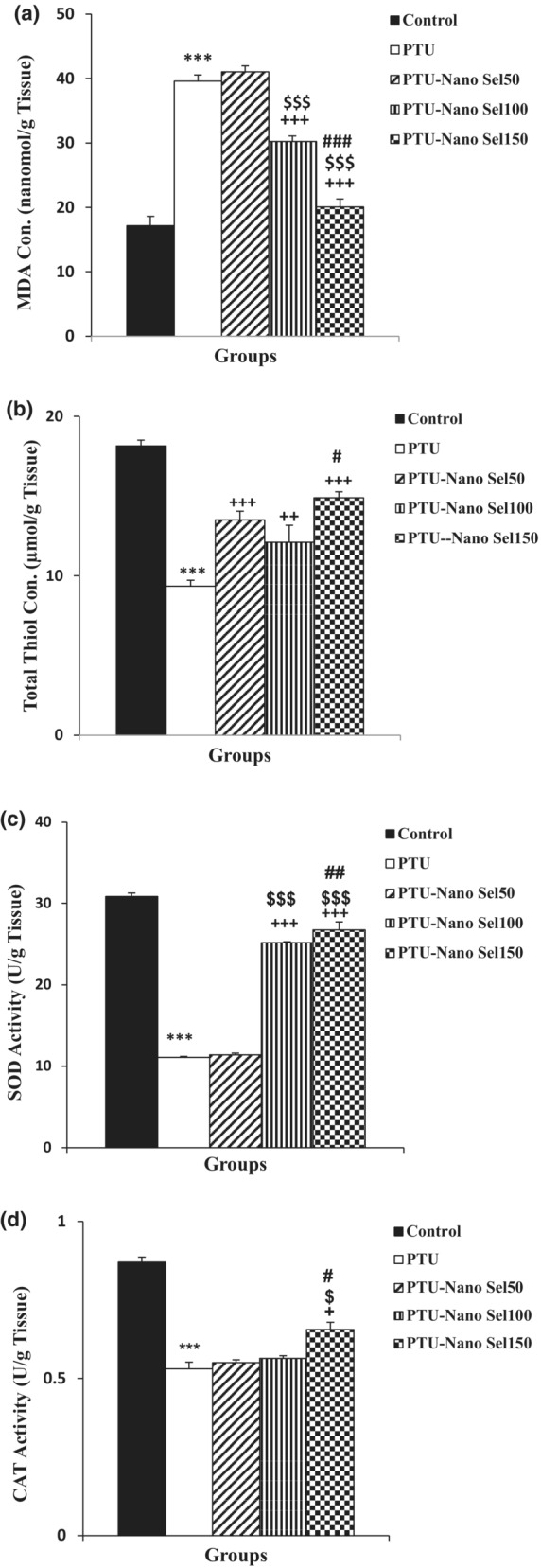
Comparison of hepatic activity, MDA (a), total thiol (b), SOD (c), and CAT (d), between experimental groups. Results were revealed as mean ± SEM. ***p < 0.001 versus control group; ^+^ *p* < 0.05, ^++^ *p* < 0.01 and ^+++^ *p* < 0.001 versus PTU group; ^$^ *p* < 0.5 and ^$$$^ *p* < 0.001 versus Nano Sel50 group and ^#^ *p* < 0.05, ^##^ *p* < 0.01 and ^###^ *p* < 0.001 versus Nano Sel100 group.

**FIGURE 6:**
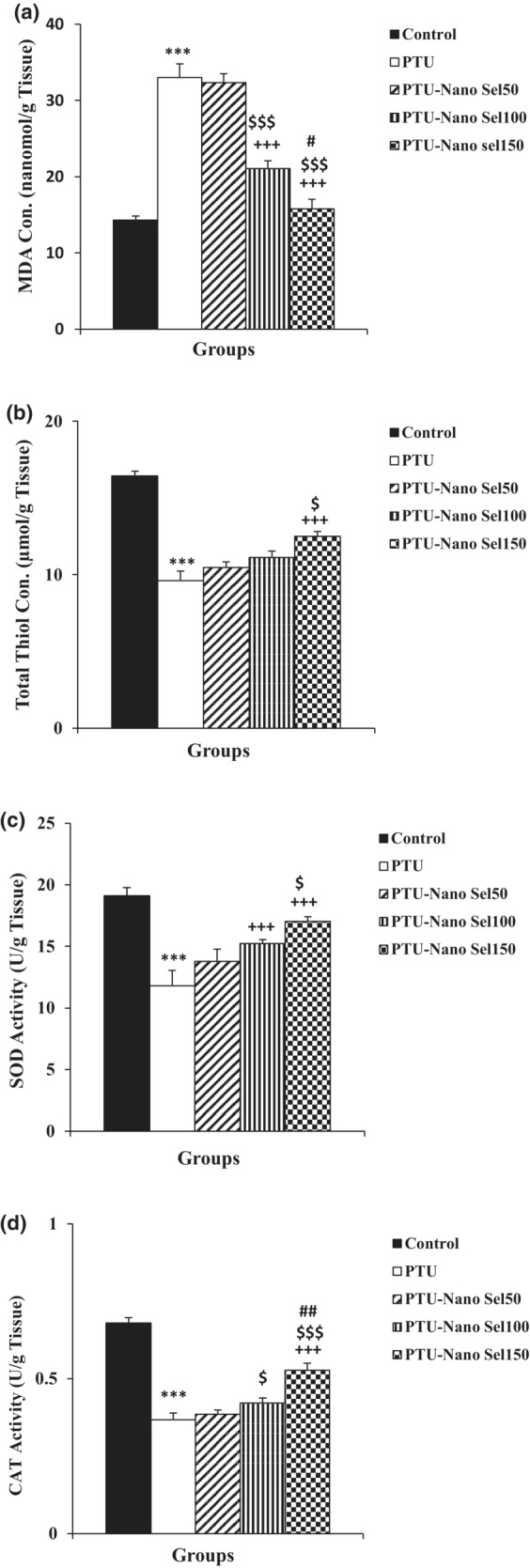
Comparison of renal activity, MDA (a), total thiol (b), SOD (c), and CAT (d), between experimental groups. Results were exhibited as mean ± SEM. ***p < 0.001 versus control group; ^+++^ *p* < 0.001 versus PTU group; ^$^ *p* < 0.05 and ^$$$^ *p* < 0.001 versus Nano Sel50 group and ^#^ *p* < 0.05 and ^###^ *p* < 0.001 versus Nano Sel100 group.

According to the biochemical findings, the accumulation of MDA in the liver and renal tissues of rats exposed to PTU was higher than in the control group (*p* < 0.001). The MDA concentration remarkably decreased in the liver and renal tissues of PTU‐Nano Sel100 and PTU‐Nano Sel150 groups compared with the PTU group (*p* < 0.001). Results indicated that the MDA concentration in PTU‐Nano Sel100 and PTU‐Nano Sel150 groups was lower than the PTU‐Nano Sel50 group (*p* < 0.001). The MDA level in the liver (*p* < 0.001) and renal (*p* < 0.05) tissues also had a remarkable decrease in the PTU‐Nano Sel150 group versus the PTU‐Nano Sel100 group (Figures 5a and 6a).

The results of total thiol demonstrated that there was considerable mitigation in the content of this antioxidant marker in liver and kidney tissues in the PTU group versus the control group (*p* < 0.001). Analysis of liver tissue findings revealed that the total thiol concentration enhanced in PTU‐Nano Sel50 (*p* < 0.001), PTU‐Nano Sel100 (*p* < 0.01), and PTU‐Nano Sel150 (*p* < 0.001) groups in comparison with PTU group. The content of total thiol in the liver tissue of the Nano Sel150 group was higher than the Nano Sel100 group (*p* < 0.05). Based on the results of kidney tissue, the total thiol level in the Nano Sel150 group was higher than the PTU group (*p* < 0.001). There was detected no significant difference in the total thiol content of renal tissue in PTU‐Nano Sel50 and PTU‐Nano Sel100 groups versus the PTU group. Data showed a noticeable increase in the total thiol content of kidney tissue in the Nano Sel150 group compared with the Nano Sel50 group (*p* < 0.05; Figures 5b and 6b).

The findings of this study determined that the activity of SOD and CAT in the liver and renal tissues lessened in the PTU group compared with the control group (*p* < 0.001). The SOD activity of hepatic and renal tissues was higher in the PTU‐Nano Sel100 and PTU‐Nano Sel150 groups with respect to the PTU group (*p* < 0.001). SOD activity of hepatic tissue in PTU‐Nano Sel100 and PTU‐Nano Sel150 groups also was higher than the PTU‐Nano Sel50 group (*p* < 0.001). The level of SOD activity of hepatic tissue in the PTU‐Nano Sel150 group was higher than in the PTU‐Nano Sel100 group (*p* < 0.01). The results also determined that the CAT activity of hepatic tissue in the PTU‐Nano Sel150 group was higher than PTU‐Nano Sel50 (*p* < 0.05). CAT activity of renal tissue in PTU‐Nano Sel100 (*p* < 0.05) and PTU‐Nano Sel150 (*p* < 0.01) groups was higher than in the PTU‐Nano Sel50 group. CAT activity of hepatic (*p* < 0.05) and renal (*p* < 0.01) tissues in the PTU‐Nano Sel150 group was higher than the PTU‐Nano Sel100 group (Figures 5c,d, 6c,d).

## 4 DISCUSSION

Hypothyroidism is a clinical condition in which the serum level of thyroid hormones is lower than the normal range. In experimental studies, one of the conventional methods for hypothyroidism induction is the use of PTU in drinking water (Erbas, 2022). In this study also, hypothyroidism was induced by adding PTU to drinking water. In plenty of animal studies, measurement of fT4 level has been used to confirm hypothyroidism (Gomes et al., 2008). In our research, hypothyroidism caused by PTU was also checked by assessing the fT4 level in the blood samples of the rats. The results indicated a significant reduction in serum level of fT4 in rats treated by PTU versus those of the control group, which confirms a hypothyroidism status; however, measuring the blood level of TSH and T3 is suggested.

Evidence also shows that hypothyroidism negatively affects hepatorenal function (Cano‐Europa et al., 2011; Dizaye & Mustafa, 2019). In addition, the enhanced blood concentration of liver enzymes, including AST, ALT, and ALP, and decreased serum levels of albumin and total protein are considered as indicators approving hepatic dysfunction (Beheshti et al., 2021). In the present study, hypothyroidism induced by PTU overturned the normal function of the hepatic and kidneys in the rats. The hepatic malfunction was obvious from increased serum levels of AST, ALT, and ALP and decreased blood concentration of albumin and total protein in the PTU group compared with the control group. These results were in agreement with the results of previous studies (Hussain et al., 2023; Song et al., 2022). Total bilirubin and direct bilirubin are also additional markers for liver function; however, they were not measured in the current study. In contrast to these findings, it was reported that hypothyroidism attenuated liver fibrosis (Bruck et al., 2007). Therefore, it seems that histopathological assessment and cellular and molecular tests could be useful for more accurate determination of liver damages resulting from PTU‐induced hyperthyroidism. High blood level of creatinine and BUN has also been reported in kidney injuries (Geshnigani et al., 2023). Increased blood levels of creatinine and BUN in the PTU group versus those of the control group also ascertained the renal disturbance resulting from hypothyroidism in the present study.

Oxidative stress takes place when the ability of the antioxidant defense system in neutralizing free radicals and ROS decrements (Beheshti et al., 2021). Oxidative injury can be a consequence of lipid peroxidation and attenuation of antioxidant effects of thiol groups in the cells (Niki, 2008). Thyroid hormones have been suggested to prevent oxidative stress via the potentiation of the antioxidant defense system (Das & Chainy, 2004). Evidence has also demonstrated that hypothyroidism enhances lipid peroxidation and stimulates the overgeneration of ROS and eventually induces oxidative stress (Das et al., 2022; Duntas, 2005). In this study, hypothyroidism also led to an elevated level of MDA and reduced the content of total thiol groups in the hepatic and renal tissues of the rats treated by PTU with respect to the animals of the control group. In addition, free radicals can be removed by antioxidant enzymes such as SOD, CAT, and GPx (Kankofer et al., 2005). It has been documented that oxidative stress‐linked hypothyroidism decreased the activity of SOD and glutathione levels in rats (Ayuob et al., 2020). Furthermore, improvement of oxidative stress and enhancement of CAT activity followed by levothyroxine in patients with primary hypothyroidism have been illustrated (Masullo et al., 2018). In line with these reports, in current research, the activity level of SOD and CAT declined in liver and kidney tissue in hypothyroid animals by PTU in comparison with the control group. Therefore, it is speculated that oxidative stress contributes to the harmful effects of PTU‐stimulated hypothyroidism in the current study.

Based on the results derived from this research, administration of all three doses of Nano Sel could restore the harmful effects of hypothyroidism on hepatic and renal tissues and normalize the function of these two vital organs in the rats. The results revealed a decreased level of AST, ALT, ALP, creatinine, and BUN and an increased concentration of albumin and total protein in the PTU‐Nano Sel groups versus the PTU group. The hepatorenal protective effect of Nano Sel was also associated with the increased blood levels of fT4 in PTU‐Nano Sel100 and PTU‐Nano Sel 150 groups in comparison with the PTU group. Nano Sel has been shown to have antioxidant and anti‐inflammatory effects (Abdou & Sayed, 2019; Hojjati Fard et al., 2022). In an animal study, Nano Sel has been shown to protect the liver against toxicity resulting from cadmium by balancing the hepatic function markers and restoring the activity of antioxidant enzymes, including CAT and GPx (Vicas et al., 2021). Nano Sel also could ameliorate the injuries resulting from inflammation and oxidative stress in the intestine of juvenile grass carp fed by a high‐fat diet (Liu et al., 2022). In another research, Nano Sel alleviated oxidative stress and inflammatory reactions and suppressed the hepatocytes apoptosis followed by Di‐(2‐ethylhexyl) phthalate (DEHP) administration in the chicken liver (Li et al., 2021). In our previous study, the administration of Nano Sel also improved cardiac fibrosis followed by hypothyroidism. This cardioprotective effect of Nano Sel was associated with the modulation of oxidative stress status in the heart and aorta tissues of the rats (Rastegar moghaddam et al., 2022). In parallel with these findings, in the current study Nano Sel especially the doses of 100 and 150 mg/kg could antagonize the malic effects of hypothyroidism on hepatic and renal tissues via restoring the oxidative stress status. Based on the data, injection of 100 and 150 mg/kg of Nano Sel lowered MDA level, raised total thiol group concentration, and fortified SOD activity in hepatic and renal tissues with respect to the PTU group. Analysis of the findings also indicated that only the dose of 150 mg/kg of Nano Sel could increase the activity of CAT in liver and kidney tissues in comparison with the PTU group. Biochemical results also demonstrated that there was no significant difference in the level of MDA in the group treated with 50 mg/kg of Nano Sel compared with the PTU group. Additionally, administration of 50 and 100 mg/kg of Nano Sel could not enhance the activity of CAT significantly when it was compared with the PTU group. Relying on these findings, it was guessed that the effects of Nano Sel probably are carried out independent of its dose. These results exhibited that the antioxidant properties of Nano Sel can be contributed to the amelioration of hepatic and renal dysfunction caused by hypothyroidism in the present study. To determine more precisely the antioxidant effects of Nano Sel on hepatic and renal injuries caused by PTU, the western blots test is also recommended to be done in future works.

## 5 CONCLUSION

In conclusion, Nano Sel could antagonize the pernicious effects of hypothyroidism on hepatic and renal function. Improving effects of Nano Sel associated with the restoration of liver and kidney function markers and modulation of oxidative stress status.

## AUTHOR CONTRIBUTIONS

Mahmoud Hosseini wrote the proposal and analyzed the data; Farimah Behehsti and Narges Marefati fulfilled the experiments and collected the data; Akbar Anaeigoudari analyzed the data, prepared the draft of the manuscript, and submitted the manuscript. All the authors revised the manuscript and confirmed it.

## FUNDING INFORMATION

This research was suported by Jiroft University of Medical Sciences.

## ETHICS STATEMENT

The study was approved by the Ethical Committee of Jiroft University of Medical Sciences, Jiroft, Iran (IR. JMU, REC. 1400. 068).

## CONFLICT OF INTEREST STATEMENT

There is no conflict of interest.
